# Dirty Utility Rooms of Hospitals in Saudi Arabia: A Multi-Regional Case Study

**DOI:** 10.3390/ijerph22040604

**Published:** 2025-04-11

**Authors:** Khalid Alkhurayji, Abdulmunim Alsuhaimi, Dalal Alshathri, Dlal Almazrou

**Affiliations:** 1 Research, Statistics, and Information Department, Saudi Central Board for Accreditation of Healthcare Institutions, Riyadh 12264, Saudi Arabia; dalshathri@cbahi.gov.sa; 2 Executive Department of Standards, Saudi Central Board for Accreditation of Healthcare Institutions, Riyadh 12264, Saudi Arabia; aalsuhaimi@cbahi.gov.sa; 3 Development of Standards and Evaluation System Section, Saudi Central Board for Accreditation of Healthcare Institutions, Riyadh 12264, Saudi Arabia; dalmazrou@cbahi.gov.sa

**Keywords:** infection control, occupational health, patient safety, quality of healthcare

## Abstract

**Background:** The dirty utility room (DUR) plays a vital role in maintaining and optimizing the safety of patients and healthcare staff. A substantial gap exists in the literature concerning the current topic in terms of empirical studies and reviews. Therefore, this study aims to shed light on the subject and provide reliable evaluations. **Methods:** A qualitative case study design (observational) was used. We included the DURs of hospitals in multiple regions of the Kingdom of Saudi Arabia/in wards and units of each hospital. To achieve data saturation, visits across wards and ICUs were conducted until no new information was retrieved. NVivo Software version 14 was used for management and analysis of the data. We used our notes to initiate codes and then created themes involving the six steps of thematic analysis for the observational study. **Results:** Among several main hospitals in the central, western, eastern, southern, and northern geographical locations in Saudi Arabia that included DURs, a total of 24 DURs were explored to capture all relevant aspects related to the observations. Considering the range of items presented in DURs, the majority of hospitals exhibited a substantial lack of equipment. There were disagreements regarding the definition of DURs and the name of DURs. The observers agreed with the practice of urine disposal, which is performed by hand. The observers from all regions mutually agreed that stool disposal methods for patients involved diapers and the cleaning of patients manually with bed sheets. Several risks of infection control were observed related to DUR design and protocols. **Conclusions:** This national observational study of DURs in Saudi Arabian hospitals revealed major inadequacies in the design, equipment, and processes that are critical for infection control and healthcare quality, emphasizing the critical necessity for standardized methods and appropriate equipment.

## 1. Introduction

The dirty utility room (DUR) is described according to the International Health Facility Guidelines (IHFG) as follows: “The Dirty Utility provides for cleaning and holding of used equipment for collection and sterilization elsewhere, disposal of clinical and other wastes and soiled linen, testing and disposing of patient specimens and decontamination and storage of patient utensils such as pans, urinals and bowls” [1]. As a result, the DUR plays a vital role in maintaining and optimizing the safety of patients and healthcare staff, as well as improving the efficiency and effectiveness of health institutions’ resources [2,3]. Most impoverished countries lack adequate physical facilities, particularly in surgical and maternity wards. As a consequence, there could be a risk of infection in hospital settings due to inadequate ventilation and air conditioning [4].

The management of hospital operation rooms and wards requires adequate facilities and functions, including support service areas [5]. Furthermore, these areas can be categorized as a functional unit, such as long-term stay or acute care. Alternatively, some hospitals categorize areas depending on patient characteristics, such as stroke patient units. Thus, each area requires a DUR to ensure proper patient care management [6]. These locations are used for the temporary storage and disposal of contaminated equipment following best practices, guidelines, and international standards; otherwise, there is a risk of infection and hazards at work if these areas are not managed properly [7].

According to the National Services Scotland (NSS), dirty utility rooms could serve up to 15 beds for better management and efficacy [8]. Additionally, the IHFG provides important standards in terms of fixtures and equipment that must be included in DURs, such as a basin, hand wash, a washer disinfector, a spray hose, a sink (size of 450 × 350 × 250D), a sink flushing rim, and washer disinfector equipment. Furthermore, the preconstruction of utility rooms must consider gaps between surfaces since these gaps can lead to the development of dust, molds, and vermin [1].

The DUR is a closed room found in healthcare institutions designed to manage human waste products in addition to the disposal of materials and disinfection of their associated items [1]. The main purpose of the DUR is to provide efficiency and effectiveness in the management of human waste [2]. Disagreements have been observed among healthcare entities in terms of establishing the definition of a DUR. For example, some healthcare providers and institutions refer to the DUR as the soiled utility room (SUR) [3]. On the other hand, some healthcare institutions refer to it as the sluice room (SR) [4]. In both definitions, the tasks and functions are the same. However, this disagreement may contribute to the misclassification and identification of required equipment in hospitals for DURs [5].

The provision of a utility room for materials and equipment that come into contact with patients makes it necessary to keep it sanitary for future patient use [6]. In such cases, the use of patient materials and equipment, as well as noncompliance with infection control protocols and regulations, puts patients at high risk of infection. These materials and pieces of equipment can transmit microorganisms, which are extremely dangerous if they are left unchecked [7]. Therefore, the DUR is specifically designed to not allow such transmission [8]. The second aspect is ensuring the safe disposal of human waste products and other contaminated items, reducing the risk to patients and workers [9]. Furthermore, benefits can be seen in terms of operational efficiency by standardizing and enforcing cleaning and disposal processes, which maintain the sanitation of the environment prior to and after patient services [10].

The prevalence and standards of DURs have not been extensively reported and documented in Saudi Arabia, and while comprehensive data on DURs are limited, the importance of essential infection control practice across Saudi Arabian hospitals is well recognized. The role of DURs is integral to infection control in Saudi hospitals. To illustrate this, previous investigations of healthcare-associated infection (HAI) prevalence across multiple-regional Saudi hospitals reported a prevalence of 6.8% for pneumonia, urinary tract infection, and bloodstream infection. These findings emphasized the critical need for properly designed and managed DURs to mitigate HAIs [11,12].

Despite the advancement and development in the healthcare system of Saudi Arabia, infection control poses multiple challenges such as HAIs, emerging infectious diseases, and resource limitations, which remain a concern in Saudi Arabian hospitals [13,14].

In light of the literature, past investigations have demonstrated that the DUR in an Intensive Care Unit (ICU) is recognized as a possible source of infection and transmission [9]. Despite earlier research, a substantial gap is evident in the literature concerning the current topic in terms of empirical studies and reviews. Therefore, this study aimed to explore this subject and provide reliable evaluations, as well as solid recommendations for improving guidelines and enhancing healthcare infection control practice and patient safety.

## 2. Materials and Methods

### 2.1. Study Design

Qualitative case study design (observation).

### 2.2. Study Area/Setting

The study setting included dirty utility rooms in hospitals of the Kingdom of Saudi Arabia/in wards and units of each hospital.

### 2.3. Study Subjects

The researchers included all hospitals that provided permission to conduct the case study. However, hospitals that were rejected and/or did not respond were excluded given that participation in this study was voluntary. The main reasons for rejection or non-response included administrative approval delays, institutional confidentiality concerns, and resource constraints that limited hospitals’ ability to engage in the study. Despite the potential selection bias, the final sample still represents major hospitals across different geographic regions. Hospitals were selected based on geographical distribution to capture regional variation and major hospitals across regions. These hospitals handle complex cases, high patient volumes, and infection control challenges, which means they represent broader infection control and healthcare practices. Furthermore, the major hospitals selected serve as regional reference centers, influencing infection control policies and practices in smaller healthcare facilities.

### 2.4. Sample Size and Sampling Technique

The researchers used the purposive sampling technique to select wards and ICUs for this study. The basis for this selection is that DURs are found in the ICU and inpatient wards. According to the literature, no consensus has been established about the optimal sample size required for a qualitative study design. However, to achieve data saturation, visits across wards and ICUs were conducted until no new information was retrieved. Saturation was reached when the last observations yielded no new information about DUR practice, equipment, and design. This approach aligns with established qualitative research guidelines.

### 2.5. Data Collection Methods, Tools, and Measurement Instructions

The researchers used observation as a way of gathering data by observing behaviors, events, and settings, followed by writing notes during the field study.

### 2.6. Data Management and Analysis Plan

NVivo software was used for data analysis and management [15]. We used our notes to initiate codes inductively, followed by the creation of themes involving the six steps of thematic analysis for the observational study [16]. Counts, ranges, percentages, and central tendency measures were analyzed. Furthermore, the data are presented in tabulation, figure, and narrative forms.

### 2.7. Data Sharing Management

All the data generated from this study will be shared upon request from the principal investigator, and the data will be appropriately stored in the document system.

### 2.8. Ethical Considerations

In medical science, ethics plays an important role when dealing with hospital data. As a result, to maintain the integrity of this case study, appropriate precautions were taken to protect patients’ privacy and confidentiality. To ensure data security, the research team members received and adhered to instructions for managing sensitive data, which were saved appropriately. The findings were disseminated, and the report was presented in an aggregate manner to maintain hospital anonymity and confidentiality. Verbal informed consent was obtained prior to the beginning of observation and/or during the taking of notes. Furthermore, the researchers ensured that any data obtained from this case study were shared with analysis units post-observation and that they could withdraw and/or remove data at any point prior to publication.

## 3. Results

Among several main hospitals in Saudi Arabia from the central, western, eastern, southern, and northern geographical locations, the DURs within wards and units from different departments, including the ICU, ER, and inpatient wards, were observed to capture current practices and provide generalizable assessments. A total of 24 DURs were observed to capture all relevant aspects related to the observations, among which 4 DURs across wards and ICUs in western hospitals were examined. Similarly, DURs in wards and ICUs were examined in hospitals located in the southern, northern, western, and eastern regions (Table 1).

### 3.1. Main Themes

**Figure 1:**
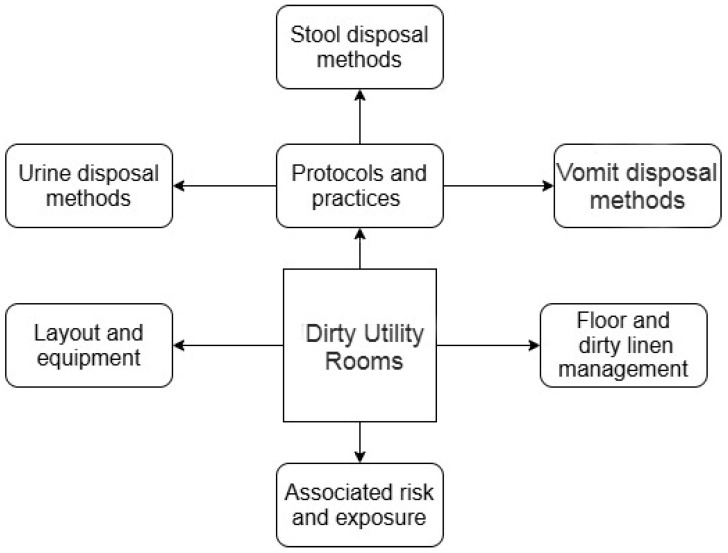
Themes and related sub-themes.

Figure 1 shows the four key themes that emerged from the data analysis, which included DUR layout and equipment; protocols and practices, with three sub-themes (urine disposal methods, stool disposal methods, and vomit disposal methods); floor and dirty linen management; and associated risk and exposure.

### 3.2. DUR Layout and Equipment

It was observed that “The rooms are mainly used to store dirty and clean linens”. The observer of central hospitals noted that regarding DUR usage, the DURs were only used for storing dirty linens and cleaning linens. Additionally, they stated that “Toilet rooms have been used as DURs to store dirty linen only, there’s no physical DUR room in the hospital”. The observer of southern hospitals demonstrated the critical need for dedicated DURs to dispose of all general waste items such as human waste and dirty liquid buckets. Moreover, according to the western hospital observer, “The absence of hand wash basin sink in the dirty utility room and absence of a deep wash sink restricts the facility’s ability to conduct comprehensive cleaning of contaminated items”. The observer highlighted that there were critical gaps in terms of the essential equipment and layout needed for the operation of the DUR to serve patients and help healthcare providers in the best way possible. However, according to the observer of the central hospital, “Hand wash basins were available in most DURs, even though some bedpan washers were out of service”. The observer also reported that some DURs had the necessary equipment and layout. However, some of them were not used. In addition, the lack of a slop hopper was evident. The DUR personnel and the observers mutually agreed that proper storage cabinets and/or shelves for cleaning items were lacking.

In addition, the room sizes varied across the regions of Saudi Arabia, and in fact, several observers stated that “Personnel can enter these rooms with restrictions”. Moreover, it was observed that “the ratio of DURs for each department was five, which found it serves more than the recommended number of patients”. Regarding the type and conceptualization of DURs, there was disagreement regarding the names used for DURs. However, the objective of all DURs was to ensure effective, safe, sanitary waste disposal and reduce the danger of infection transmission. The types of DURs differ depending on a variety of factors, including the volume of waste generated in wards or units, the size of the institution, and the regional regulatory authority. Each type of DUR is built with the specific needs of the wards and/or units in mind, regardless of technology or plan variances, and tailored to a variety of personnel, including healthcare practitioners, facility planners, or simply people interested in understanding the complexities of healthcare operations [17]. More than 20 pieces of equipment can be observed in DURs, demonstrating the importance of utility services being available in this space. Examples include disposing of paper towels, eliminating any unpleasant odors, offering temporary storage, and disinfecting reusable bedpans or urine bottles (Table 2) [17].

### 3.3. Protocols and Practices

#### 3.3.1. Urine Disposal Methods

The observer of hospitals from eastern regions stated that “*The most commonly used disposal method involved the use of reusable urinal bottles for a single patient, which are emptied and washed by hand. Additionally, intubation urinal bags are emptied in two steps: first, by urinal bottles, followed by emptying by hand, with all these procedures being performed inside the patient’s toilet without wearing PPE*”. Additionally, the observer of hospitals from western regions stated that “*single-use urinal bottles were emptied by hand and disposed in medical/general waste bins*”. Observers jointly agreed that urine disposal practices can involve these two procedures. However, one observer stated that “*during the emptying of the urinal bottles, some healthcare providers used PPE for certain ICU patients*”. In the manual handling of urine, when poured or splashed, specific microscopic droplets can result in aerosolization and potential pathogen transmission, potentially carrying bacteria, viruses, and fungi, which lead to an increased risk of inhalation and surface contamination.

#### 3.3.2. Stool Disposal Methods

The observers from all of the regions mutually agreed that stool disposal methods for patients were provided through the use of diapers and manually cleaning patients with bed sheets. According to the observer of the central region, “Patient is cleaned manually with cleaning paper; the dirty items are placed in a bag and thrown in medical/general waste bins”, and the observer from the east highlighted that “Patient is cleaned manually with cleaning paper; the dirty items are placed in a bag and thrown in medical/general waste bins”.

#### 3.3.3. Vomit Disposal Methods

According to several observers from different regions, “The hospital provides a spill kit for managing vomit; if vomit reaches the floor, the same kits are used for cleanup”. The concern of observers highlights that a spill kit is only used in the case of vomit reaching the floor. However, this is not the case if the vomit remains in the patient’s bed.

### 3.4. Floor and Dirty Linen Management

According to the observers, “*Cleaning floor buckets are emptied in the normal janitors’ room and dirty linen can be temporarily kept inside the DUR*”. The observers agreed with the current practice for floor and dirty linen management.

### 3.5. Associated Risk and Exposure

According to observers from the northern and western regions, “Storing dirty linens in a toilet room can pose several challenges and risks, including cross-contamination”. The observers highlighted that a toilet room is often a high-risk area for the transmission of bacteria and other pathogens. In addition, storing dirty linens in the same space as toilets increases the risk of cross-contamination between the linens and bathroom surfaces, which could contribute to the transmission of infections, particularly in a hospital environment. Redesigning DURs to be placed in easily accessible locations and providing entry areas, soiled equipment processing, hand hygiene stations, storage areas, and ventilation systems could mitigate these risks. Nonetheless, converting toilets into fully functional DURs can improve waste segregation and infection control and optimize hygiene measures.

Furthermore, one observer noted that “Proper infection control protocols require that soiled linens be handled and stored in areas designed for that purpose, which are typically equipped with the right hygiene and sanitation measures”. The observer reported that a toilet room is not designed to meet these infection control standards and that its use for storing dirty linens might not be compliant with healthcare regulations. Moreover, in terms of airborne contaminants, the observers mutually agreed that “Toilets can release aerosols when flushed, which may contain harmful microorganisms”. These observers highlighted that storing dirty linen in the same space as toilets could potentially lead to contamination of the linens, which might then be transferred to patients or other areas of the hospital.

The observers from the central and southern regions stated that “Storing soiled linen in a toilet room might create unpleasant odors in the surrounding areas, which can negatively affect both patient and staff comfort”. The observers highlighted that using a toilet room to store dirty linen in a hospital is not ideal and could pose risks in terms of infection control, hygiene, and patient safety; thus, hospitals that use toilet rooms to store dirty linen should redesign their DURs. Additionally, one of the observers at the north hospital stated that “Operating the DUR for the ICU outside the unit reflects the urgency to redesign the DURs to improve infection control and patient safety”.

## 4. Discussion

This national observational study highlighted the current practices regarding DURs in several regions of Saudi Arabia, reflecting the generalizability of our findings regarding DURs in ICUs and wards within hospitals. The absence of proper design, protocols, and equipment could impact infection control and the quality of healthcare for the population in hospital settings. The majority of protocols and current practices vary, and there is a need for the development of standards to improve the current status of DUR utilization in hospitals.

As observed across different Saudi Arabian regions, the absence of a deep wash sink in DURs was evident. A lack of this equipment restricts a facility’s ability to conduct comprehensive cleaning of contaminated items, which is necessary to uphold infection control standards [18]. Notably, the manual handling of contaminated waste in this manner increases the likelihood of healthcare-associated infections (HAIs), posing severe risks not only to staff but also to vulnerable patient populations [19]. In this observation, the absence or malfunctioning of the bedpan washer necessitates end-users to manually empty and clean bedpans, frequently without the appropriate use of PPE or adherence to standardized infection control methods. Furthermore, each DUR should have a slop hopper to empty dirty liquid from the floors of the ward, ICU, ER, and OR and not allow the dirty liquid to be moved to the janitor’s room. This study’s findings illustrate that due to the lack of a slop hopper/slop sink in many DURs, buckets containing dirty liquid are being emptied in the janitor’s room [20]. According to our study, dedicated slop hoppers/slop sinks in dirty utility rooms are lacking, despite the fact that the Centers for Disease Control and Prevention (CDC), World Health Organization (WHO), and ISO 14698 standards reflect the critical need for designated sinks for biohazardous waste in clinical settings to prevent pathogen transmission. The WHO underscores the importance of appropriate waste management facilities to protect healthcare workers and patients alike [21,22,23]. Without a slop sink, healthcare staff are often forced to dispose of biohazardous waste in janitorial or general-purpose sinks that are not engineered for handling infectious materials. This practice increases the risk of the aerosolization and splashing of contaminated fluids, leading to potential environmental contamination and pathogen spread in nonclinical areas.

Implementing certain equipment, like deep wash sinks, can be costly for each DUR, ranging from USD 2000 to USD 5000 [24]. Despite the cost, long-term benefits can be achieved in that the fewer the HAIs, the greater capacity the hospital will gain, and the more total hospital costs will be reduced [25].

In comparison to other countries, Australian healthcare hospitals are designed according to guidelines that specify DUR to be centrally located for easy access to the inpatient area and direct access to the unit [26], while the Health Service Executive (HSE) in Ireland provides standards and guidance for staff, including protocols for the maintenance and operation of dirty utility rooms [27]. However, in Saudi Arabia, practices and protocols are inconsistent, and there is a need to standardize protocols and adhere to best practices. The inadequate designs, equipment, and protocols pose significant infection risks in terms of aerosolization and airborne contamination, water source contamination, inadequate cleaning and maintenance, improper waste and linen handling, and design flaws leading to cross-contamination.

According to several international standards, such as the IFHG, NHS, WHO, and CDC, the absence of a dedicated cleaning storage area with stainless steel shelving and cabinets in dirty utility rooms specifies the need for contamination-resistant, easily sanitized storage for cleaning supplies to prevent cross-contamination. Stainless steel, which is recommended for its durability and ease of sterilization, is essential for minimizing HAIs. Without such storage, improperly stored cleaning tools become vectors for pathogen transmission, increasing the risk of HAIs and potential severe outcomes for patients and staff [18,20,23].

According to the IHFG [18], DURs should serve up to 15 beds. However, in our study, this ratio is not the same as that in the majority of hospitals; that is, one DURs is used for more than this number of beds, highlighting the need to redesign these rooms. All dirty utility/disposal rooms should undergo a minimum of six air changes per hour (extraction only) and be maintained at a negative pressure (−5 Pa) compared to surrounding areas [28].

Manual emptying of urinals can generate aerosols containing microorganisms. Studies have shown that bodily fluids containing pathogens, such as *Escherichia coli*, *Clostridium difficile*, and *Staphylococcus aureus*, can become airborne during handling [29]. These aerosols can remain suspended in the air and contaminate surfaces, increasing the risk of HAIs. Pathogens can easily spread to surfaces and equipment in and around a dirty utility room due to splashing during manual emptying. Research has demonstrated that contaminated surfaces serve as reservoirs for pathogens and are a primary mode of HAI transmission in hospitals [30]. Urinal surfaces and handling containers may act as fomites, which transfer pathogens. Several studies have shown that fomites can contribute to the transmission of infectious diseases in healthcare environments [31]. Manual cleaning generates aerosols, particularly when scrubbing and washing are involved. These aerosolizers can disseminate pathogens through the air, leading to infection and airborne transmission. Studies indicate that certain pathogens, such as *S. aureus* and *C. difficile*, can be aerosolized during manual cleaning, increasing the likelihood of HAIs [29]. These pathogens can stay on surfaces or be inhaled, resulting in infection of both healthcare providers and patients. The prolonged contact from diaper changing and exposure to moisture can lead to skin maceration, which weakens the skin barrier and increases the chance of infections such as *S. aureus* and *Candida albicans*. Previous studies have shown that patients in diapers are at greater risk of developing pressure ulcers and associated infections than those using properly sanitized bedpans [32].

The findings of our study illustrate the need for a standardized protocol and proper design of DURs in hospitals in Saudi Arabia. Addressing concerns about infection control practices and deficiencies can improve patient safety and quality. Emphasizing the importance of managing human waste can lead to a reduction in infection control practices. Previous studies demonstrate that proper implementation of DUR and waste management can reduce HAIs [33]. Furthermore, HAI costs to U.S. hospitals range from USD 28.4 billion to USD 45 billion annually [34]. While the estimated expenses for constructing DURs range from USD 150 to USD 300 per square foot depending on the equipment and the features added [35,36]. However, the cost savings from infection prevention, operational efficiency, and compliance outweigh the expenses [37,38].

Some potential policy changes and interventions that could standardize DUR infrastructure and protocols include the specification of the requirements of DUR layout, size, and equipment, as well as DUR-specific training for healthcare providers and cleaning staff in Saudi Arabia. Nonetheless, annual certification in DUR handling protocols and inspections to check compliance with DUR standardization and protocols should be implemented.

Future research should focus on conducting long-term studies to assess improvements in DURs over time; moreover, comparative studies are encouraged to identify the difference between DUR designs and protocols across various regions. In addition, interventional studies should be conducted to improve DURs and measure their effectiveness in reducing HAIs. Moreover, studies in the context of economic impact can demonstrate how healthcare costs and patient outcomes can be improved, in particular, quantitative analyses of DUR efficiency and cost–benefit studies on their impact on infection control.

While this study includes hospitals in various regions of the Kingdom of Saudi Arabia, the findings may not reflect the actual current status of their DURs given that potential biases in data collection that emerge in observational studies could affect the accuracy of the findings. In addition to the variability in practices, providing one-size-fits-all solutions might be challenging. Despite this limitation, this study provided a thorough analysis from different perspectives across multiple regions by offering valuable insights and areas for improvement. This national study addresses critical public health concerns, making its history relevant and impactful. In addition, this study lays the groundwork for future investigations and research aiming to improve DURs and reduce HAIs.

## 5. Conclusions

This national observational study of DURs in Saudi Arabian hospitals revealed major inadequacies in the design, equipment, and procedures of DURs that are critical for infection control and healthcare quality. The lack of critical equipment, such as deep wash sinks, slop hoppers, and bedpan washers, along with poor waste management procedures, increases the risk of HAIs for both personnel and patients. The study results provide a comprehensive evaluation of DURs in Saudi Arabian hospitals, serving as a foundation for the development of standardized guidelines that align with national and international practices.

This study highlights the critical necessity for standardized methods and appropriate equipment in DURs that adhere to best practices and guidelines. Implementing these criteria can considerably minimize the risk of HAIs, increase patient safety, and improve overall healthcare quality.

## Figures and Tables

**Table 1:** Distribution of wards and ICUs across Saudi Arabian hospitals.

Variables	Number of Wards	Number of ICUs
	N (%)	N (%)
Hospital Location		
Central	4 (20.0)	4 (20.0)
Southern	2 (10.0)	2 (10.0)
Northern	2 (10.0)	2 (10.0)
Western	2 (10.0)	2 (10.0)
Eastern	2 (10.0)	2 (10.0)
Total	20 (100)	20 (100)
Hospital type		
Primary	0 (0.0)	0 (0.0)
Secondary	6 (30.0)	6 (30.0)
Tertiary	14 (70.0)	14 (70.0)
Total	20 (100)	20 (100)
Bed Size		
Small (<100 beds)	4 (20.0)	4 (20.0)
Medium (100–300 beds)	6 (30.0)	6 (30.0)
Large (>300 beds)	10 (50.0)	10 (50.0)
Total	20 (100)	20 (100)

**Table 2:** Types of DURs used in hospitals and healthcare institutes.

Dirty Utility Rooms	Description	Essential Equipment	Central	North	West	East	South
General Sluice Room	Commonly found in hospitals and nursing homes, this type of room is equipped with basic necessities such as flushers disinfectors, macerators, sinks, and storage areas. It caters to inpatients’ regular needs, handling solid and liquid waste.	Bedpan washer disinfectors, medical waste macerators, handwashing basin, clinical waste bins, handwashing sinks with elbow/pedal control, storage cabinets, and shelving units	☑	☑	☑	☑	☑
Maternity Sluice Room	In maternity wards, these rooms are designed to handle postnatal waste. They might have specialized equipment to deal with items like placenta buckets.	Bedpan washer disinfectors, medical waste macerators, handwashing basins, clinical waste bins, handwashing sinks with elbow/pedal control, storage cabinets, and shelving units	⊠	⊠	⊠	⊠	⊠
Isolation Sluice Room	These are attached to isolation wards where patients with highly infectious diseases are housed. The design and equipment in these rooms ensure that waste is handled and disposed of to minimize the risk of disease transmission.	Bedpan washer disinfectors, medical waste macerators, handwashing basins, clinical waste bins, handwashing sinks with elbow/pedal control, storage cabinets, shelving units, automated touchless disinfection dispensers, washer disinfector machines, air filtration, and UV-C light disinfection	☑	⊠	☑	☑	☑
Pediatric Sluice Room	Located in children’s wards, these rooms may contain smaller equipment or items tailored to pediatric care needs. These DURs are essential for infection control in vulnerable children.	Bedpan washer disinfectors, medical waste macerators, handwashing basins, clinical waste bins, handwashing sinks with elbow/pedal control, storage cabinets, shelving units, diaper disposal units (odor-controlled), and infant pulp product macerators	⊠	⊠	⊠	⊠	⊠
Portable or Mobile Sluice Room	A relatively new concept, these modular units can be quickly set up in emergencies or during outbreaks where a rapid response is needed. They are often deployed in field hospitals or disaster zones.	Compact bedpan washer-disinfectors, portable macerators, slop hopper alternatives, sealed foot-operated waste bins, portable hand hygiene stations, and battery-operated or solar-powered ventilation	⊠	⊠	⊠	⊠	⊠
Specialized Sluice Rooms for Outpatient Procedures	In settings like dialysis centers, where there is a need to handle specific types of waste, specialized sluice rooms might be set up. Particularly in high-turnover outpatient clinics and diagnostic units	Bedpan washer disinfectors, medical waste macerators, handwashing basins, clinical waste bins, handwashing sinks with elbow/pedal control, storage cabinets, shelving units high-capacity disposable suction liner systems, automated disinfection units, and automated waste compactors	⊠	⊠	⊠	⊠	⊠
Elderly Care Sluice Room	Often found in long-term care facilities or nursing homes, these rooms might have equipment tailored to the needs of elderly patients, focusing especially on incontinence management and the vulnerability of elderly patients, those with weak immune systems, and mobility issues.	Bedpan washer disinfectors, medical waste macerators, handwashing basins, clinical waste bins, handwashing sinks with elbow/pedal control, storage cabinets, shelving units, adult diaper and incontinence pad disposal units, macerators for adult hygiene products, and odor-controlled waste bins with automatic sealing	⊠	⊠	⊠	⊠	⊠
Mortuary Sluice Room	Situated in mortuaries or post-mortem rooms, these sluice rooms handle the specific needs related to deceased patients, such as biological waste and fluids associated with deceased patients.	Bedpan washer disinfectors, medical waste macerators, handwashing basins, clinical waste bins, handwashing sinks with elbow/pedal control, storage cabinets, shelving units, pathological waste disposal units, and heavy-duty macerators for mortuary-specific waste	⊠	⊠	⊠	⊠	⊠

⊠ = Not found; ☑ = found.

## Data Availability

The data presented in this study are only available upon request from the corresponding author due to privacy reasons.
